# Supplementary material for: Identification of Novel phoP-phoQ Regulated Genes that Contribute to Polymyxin B Tolerance in Pseudomonas aeruginosa
Source: Microorganisms. 2021 Feb 9;9(2):344. doi: 10.3390/microorganisms9020344 (PMC7916210; doi:10.3390/microorganisms9020344)
Supplement: Supplementary file 1 [file microorganisms-09-00344-s001.zip › Supplementary materials/Fig S1-S2.docx]

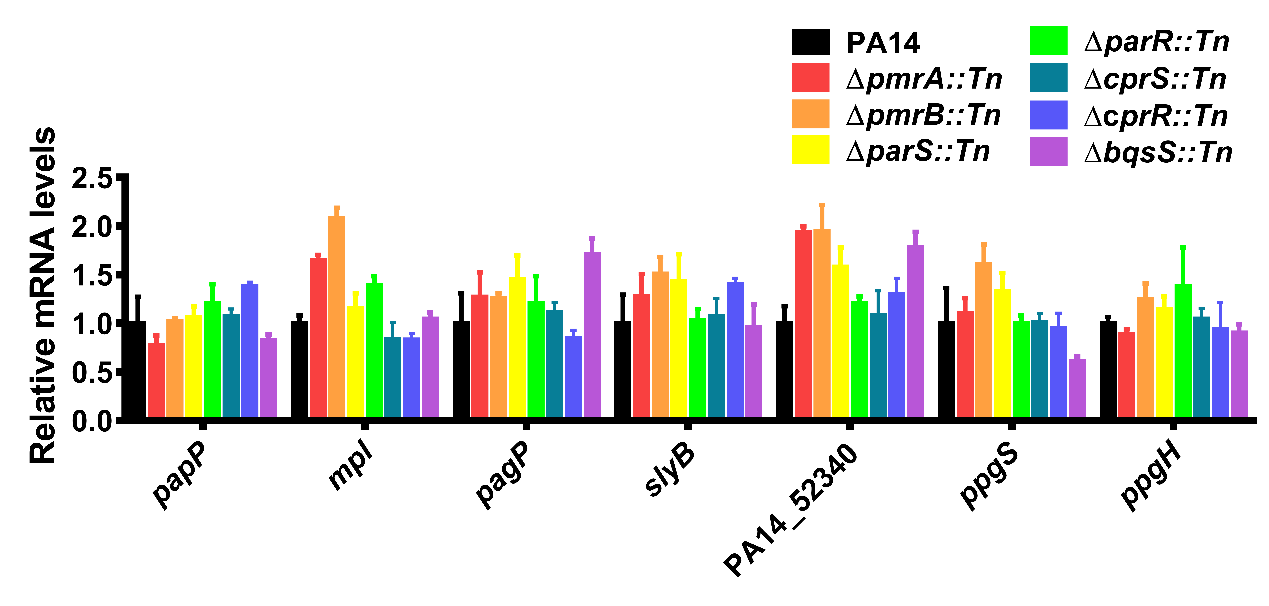


**Fig S1. Relative mRNA levels of PhoP-PhoQ-related target genes in other TCS mutants.** The wild type PA14, the *pmrA*::Tn, *pmrB*::Tn, *parS*::Tn, *parR*::Tn, *cprS*::Tn, c*prR*::Tn and *bqsS*::Tn mutants were grown in LB to an OD_600_ of 1.0. Total RNAs of the bacteria were isolated and mRNA levels of *papP*, *mpl*, *pagP*, *slyB*, PA14_52340, *ppgS* and *ppgH* were determined by quantitative real time PCR. Data represents the mean ± standard deviation from two samples.

**
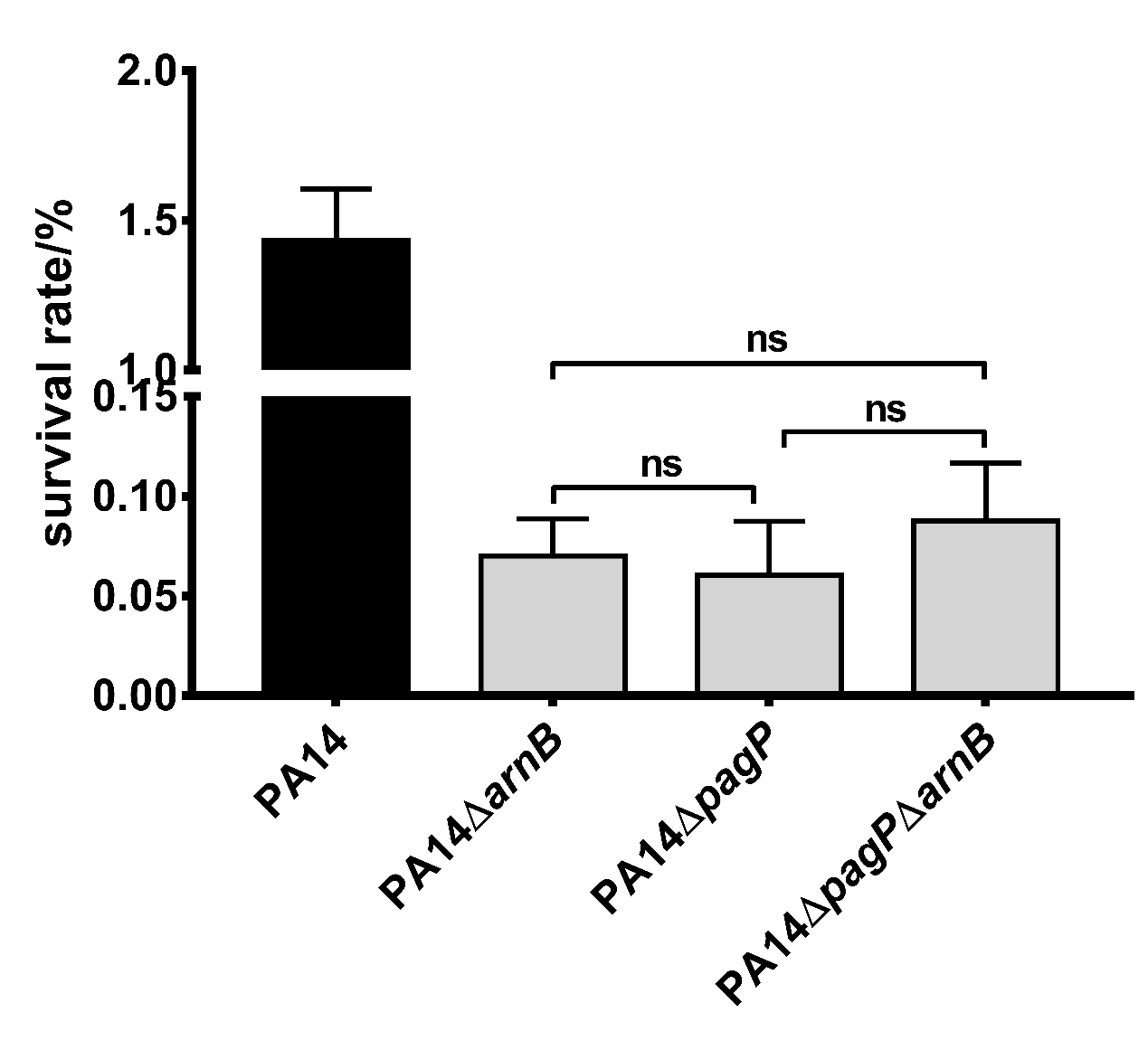
**

***

**Fig S2. Roles of *pagP* and *arnB* in the bacterial tolerance to polymyxin B.** Wild type PA14 and the indicated mutant strains was grown in LB to an OD_600_ of 1.0. After treatment with 0.78 μg/mL polymyxin B at 37 ºC for 2.5 h the bacterial survival rates were determined by serial dilution and plating. The data shown represent results from three independent experiments. ns, not significant; ***, p < 0.001 compared to the other strains by Student’s t-test.
