# Supplementary material for: Identification of Novel phoP-phoQ Regulated Genes that Contribute to Polymyxin B Tolerance in Pseudomonas aeruginosa
Source: Microorganisms. 2021 Feb 9;9(2):344. doi: 10.3390/microorganisms9020344 (PMC7916210; doi:10.3390/microorganisms9020344)
Supplement: Supplementary file 1 [file microorganisms-09-00344-s001.zip › Supplementary materials/Table S2 - Bacterial strains and plasmids.docx]

**Table S2.** Bacterial strains and plasmids used in this study.

| **Strain or plasmid** | **Description** | **Source of reference** |
| --- | --- | --- |
| **strains** |  |  |
| DH5α | F^̶^ ϕ 80d*lacZ*∆M15 *endA1 recA1 hsdR17*(r_K_^̶^ m_K_^+^) *supE44 thi-1 relA1* ∆(*lacZYA-argF*)*U169 gyrA96 deoR* | TransGen |
| S17-1 | RP4-2 Tc::Mu Km::Tn*7* Tp^r^ Sm^r^ Pro Res^̶^ Mod^+^ | (1) |
| BL21 | F^-^ *omp*T *hsd*S(r_B_^-^ m_B_^-^) dcm^+^ Tet^r^ *gal*λ(DE3) *end*A [*arg*U *pro*L Cam^r^] | Stratagene |
| PA14 | Wild type *P. aeruginosa* strain | (1) |
| PA14_11960::Tn | PA14 with a transposon inserted at PA14_11960 | (2) |
| PA14_11970::Tn | PA14 with a transposon inserted at PA14_11970 | (2) |
| PA14_11980::Tn | PA14 with a transposon inserted at PA14_11980 | (2) |
| PA14_46900::Tn | PA14 with a transposon inserted at PA14_46900 | (2) |
| PA14_50740::Tn | PA14 with a transposon inserted at PA14_50740 | (2) |
| PA14_52340::Tn | PA14 with a transposon inserted at PA14_52340 | (2) |
| PA14_52350::Tn | PA14 with a transposon inserted at PA14_52350 | (2) |
| PA14_52370::Tn | PA14 with a transposon inserted at PA14_52370 | (2) |
| *pmrA*::Tn | PA14 with a transposon inserted at *pmrA* | (2) |
| *pmrB*::Tn | PA14 with a transposon inserted at *pmrB* | (2) |
| ΔPA14_11960 | PA14 with PA14_11960 gene deleted | This study |
| ΔPA14_11970 | PA14 with PA14_11970 gene deleted | This study |
| ΔPA14_46900 | PA14 with PA14_46900 gene deleted | This study |
| ΔPA14_50740 | PA14 with PA14_50740 gene deleted | This study |
| ΔPA14_52350 | PA14 with PA14_52350 gene deleted | This study |
| ΔPA14_52370 | PA14 with PA14_52370 gene deleted | This study |
| Δ5 | PA14 with PA14_11960, PA14_11970, PA14_50740, PA14_52350 and PA14_52370 gene deleted | This study |
| Δ6 | Δ5 with PA14_46900 gene deleted | This study |
| Δ*phoP* | PA14 with *phoP* gene deleted | This study |
| Δ*phoQ* | PA14 with *phoQ* gene deleted | This study |
| Δ*phoP*Δ*phoQ* | PA14 with *phoP* and *phoQ* gene deleted | This study |
| Δ*arnB* | PA14 with *arnB* gene deleted | This study |
| ΔPA14_46900Δ*arnB* | PA14 with PA14_46900 and *arnB* gene deleted | This study |
| PA14/pUCP20-*phoP*-Flag | PA14 with Flag-tagged *phoP* gene | This study |
| PA14/vector | PA14 with pUCP20 empty vector | This study |
| ΔPA14_11960/vector | ΔPA14_11960 with pUCP20 empty vector | This study |
| ΔPA14_11970/vector | ΔPA14_11970 with pUCP20 empty vector | This study |
| ΔPA14_46900/vector | ΔPA14_46900 with pUCP20 empty vector | This study |
| ΔPA14_50740/vector | ΔPA14_50740 with pUCP20 empty vector | This study |
| ΔPA14_52350/vector | ΔPA14_52350 with pUCP20 empty vector | This study |
| ΔPA14_52370/vector | ΔPA14_52370 with pUCP20 empty vector | This study |
| ΔPA14_11960/ PA14_11960 | Overexpression of PA14_11980 in ΔPA14_11960 mutant | This study |
| ΔPA14_11970/ PA14_11970 | Overexpression of PA14_11970 in ΔPA14_11970 mutant | This study |
| ΔPA14_46900/ PA14_46900 | Overexpression of PA14_46900 in ΔPA14_46900 mutant | This study |
| ΔPA14_50740/ PA14_50740 | Overexpression of PA14_50740 in ΔPA14_50740 mutant | This study |
| ΔPA14_52350/ PA14_52350 | Overexpression of PA14_52350 in ΔPA14_52350 mutant | This study |
| ΔPA14_52370/ PA14_52370 | Overexpression of PA14_52370 in ΔPA14_52370 mutant | This study |
|  |  |  |
| **Plasmids** |  |  |
| pUCP20 | Shuttle vector between *E. coli* and *P. aeruginosa*; Amp^r^ | (3) |
| pET28b | Expression vector, Kan^r^ | Novagen |
| pEX18Tc | Gene knockout vector; Tc^r^ | This study |
| pUCP20-*phoP*-Flag | *phoP* coding sequence with its own promoter and C-terminal Flag tag in pUCP20; Amp^r^ | This study |
| pUCP20-PA14_11960 | PA14_11960 gene from PA14 in pUCP20; Amp^r^ | This study |
| pUCP20-PA14_11970 | PA14_11970 gene from PA14 in pUCP20; Amp^r^ | This study |
| pUCP20-PA14_46900 | PA14_46900 gene from PA14 in pUCP20; Amp^r^ | This study |
| pUCP20-PA14_50740 | PA14_50740 gene from PA14 in pUCP20; Amp^r^ | This study |
| pUCP20-PA14_52350 | PA14_52350 gene from PA14 in pUCP20; Amp^r^ | This study |
| pUCP20-PA14_52370 | PA14_52370 gene from PA14 in pUCP20; Amp^r^ | This study |
| pUCP20*lacZ*Ω | Promoterless *lacZ* fusion vector; Amp^r^ | This study |
| P_PA14_11970_-*lacZ*Ω | PA14_11970 promoter of PA14 fused to promoterless *lacZ* on pUCP20*lacZ*Ω; Amp^r^ | This study |
| P_PA14_46900_-*lacZ*Ω | PA14_46900 promoter of PA14 fused to promoterless *lacZ* on pUCP20*lacZ*Ω; Amp^r^ | This study |
| P_PA14_50740_-*lacZ*Ω | PA14_50740 promoter of PA14 fused to promoterless *lacZ* on pUCP20*lacZ*Ω; Amp^r^ | This study |
| P_PA14_52350_-*lacZ*Ω | PA14_52350 promoter of PA14 fused to promoterless *lacZ* on pUCP20*lacZ*Ω; Amp^r^ | This study |
| pEX18-PA14_11960 | PA14_11960 gene deletion on pEX18Tc; Tc^r^ | This study |
| pEX18-PA14_11970 | PA14_11970 gene deletion on pEX18Tc; Tc^r^ | This study |
| pEX18-PA14_46900 | PA14_46900 gene deletion on pEX18Tc; Tc^r^ | This study |
| pEX18-PA14_50740 | PA14_50740 gene deletion on pEX18Tc; Tc^r^ | This study |
| pEX18-PA14_52350 | PA14_52350 gene deletion on pEX18Tc; Tc^r^ | This study |
| pEX18-PA14_52370 | PA14_52370 gene deletion on pEX18Tc; Tc^r^ | This study |
| pEX18-*phoP* | *phoP* gene deletion on pEX18Tc; Tc^r^ | This study |
| pEX18-*phoQ* | *phoQ* gene deletion on pEX18Tc; Tc^r^ | This study |
| pEX18-*phoPQ* | *phoP* and *phoQ* gene deletion on pEX18Tc; Tc^r^ | This study |
| pEX18-*arnB* | *arnB* gene deletion on pEX18Tc; Tc^r^ | This study |
| pET28b-*phoP*-His | 6 x His-tagged *phoP* gene on pET28b; Kan^r^ | This study |

1. Li M.; Long Y.; Liu Y.; Liu Y.; Chen R.; Shi J.; Zhang L.; Jin Y.; Yang L.; Bai F.; Jin S.; Cheng Z.; Wu W. HigB of *Pseudomonas aeruginosa* enhances killing of phagocytes by up-regulating the type III secretion system in ciprofloxacin induced persister cells. *Front. Cell. Infect. Microbiol.* **2016**, *6*, 125. [https://doi: 10.3389/fcimb.2016.00125](https://doi:%2010.3389/fcimb.2016.00125).
2. Tan H.; Zhang L.; Chen R.; Liu C.; Weng Y.; Bai F.; Cheng Z.; Jin S.; Wu W. DeaD contributes to *Pseudomonas aeruginosa* virulence in a mouse acute pneumonia model. *FEMS Microbiol. Lett.* **2016**, *363*, fnw227. <https://doi.org/10.1093/femsle/fnw227>.
3. Tian Z.; Cheng S.; Xia B.; Jin Y.; Bai F.; Cheng Z.; Jin S.; Liu X.; Wu W. *Pseudomonas aeruginosa* ExsA regulates a metalloprotease, ImpA, that inhibits phagocytosis of macrophages. *Infect. Immun.* **2019**, *87*, e00695-19. <https://doi.org/10.1128/IAI.00695-19>.
