# Supplementary material for: Identification of Novel phoP-phoQ Regulated Genes that Contribute to Polymyxin B Tolerance in Pseudomonas aeruginosa
Source: Microorganisms. 2021 Feb 9;9(2):344. doi: 10.3390/microorganisms9020344 (PMC7916210; doi:10.3390/microorganisms9020344)
Supplement: Supplementary file 1 [file microorganisms-09-00344-s001.zip › Supplementary materials/Table S3 - primers.docx]

**Table S3.** Primers used in this study.

| **Primer^a^** | **Sequence 5’–3’^b^** | | **Source** |  |  |
| --- | --- | --- | --- | --- | --- |
| **Cloning of up- and downstream fragments for in-frame deletion** | |  |  |  |  |
| PA14_11960UF | CCG GAATTC ACCAGACGTACCCGGTGTAAT (EcoR I) | | This study |  |  |
| PA14_11960UR | TCC CCCGGG GGAAGGCGATGAGGAAGATG (Sma I) | | This study |  |  |
| PA14_11960DF | TCC CCCGGG TGTTGCCGGTCTGCGTG (Sma I) | | This study |  |  |
| PA14_11960DR | CCC AAGCTT CCTCAACCTGCGGCGTAT (Hind III) | | This study |  |  |
| PA14_11970UF | CCG GAATTC CACGCTCGGCTGGAACA (EcoR I) | | This study |  |  |
| PA14_11970UR | CGC GGATCC CAGGGCAAACTAAGGATGGG (BamH I) | | This study |  |  |
| PA14_11970DF | CGC GGATCC AGGACCCGCACCTGCAA (BamH I) | | This study |  |  |
| PA14_11970DR | CCC AAGCTT CCGCTGGGGAAGCTGAA (Hind III) | | This study |  |  |
| PA14_46900UF | TCC CCCGGG CGACCTCGAAGATGCCG (Sma I) | | This study |  |  |
| PA14_46900UR | CGC GGATCC AGACGCTGGTCTGCAGGTAC (BamH I) | | This study |  |  |
| PA14_46900DF | CGC GGATCC AACCTCGGCCTGCGTCT (BamH I) | | This study |  |  |
| PA14_46900DR | CCC AAGCTT GTTGCGCGCTCGTCAGG (Hind III) | | This study |  |  |
| PA14_50740UF | CCG GAATTC CGAACAGGTGGCGCAAC (EcoR I) | | This study |  |  |
| PA14_50740UR | TCC CCCGGG TGGCGGTGAAGGAAGCA (Sma I) | | This study |  |  |
| PA14_50740DF | TCC CCCGGG AGCGCGTGCGCATCC (Sma I) | | This study |  |  |
| PA14_50740DR | CGC GGATCC GCCAGAAATGGAAGGGAAACT (BamH I) | | This study |  |  |
| PA14_52350UF | CCG GAATTC TCCGTTCGCTCGCATCAT (EcoR I) | | This study |  |  |
| PA14_52350UR | CGC GGATCC TGGCGGTAGGCGGTAATC (BamH I) | | This study |  |  |
| PA14_52350DF | CGC GGATCC CGGCCTGACTGGATTGGT (BamH I) | | This study |  |  |
| PA14_52350DR | CCC AAGCTT GTTTCGCGCCCCACTTC (Hind III) | | This study |  |  |
| PA14_52370UF | CCG GAATTC CGCCCTGTACGACCCGA (EcoR I) | | This study |  |  |
| PA14_52370UR | CGC GGATCC GGCGGCGACCAGAAAAC (BamH I) | | This study |  |  |
| PA14_52370DF | CGC GGATCC TCCCTGGCGAAGAAGATGC (BamH I) | | This study |  |  |
| PA14_52370DR | CCC AAGCTT CGAAGACAGCCCACGGG (Hind III) | | This study |  |  |
| *phoP*UF | CCG GAATTC GTCGGGTTGTTCGTTCATTG (EcoR I) | | This study |  |  |
| *phoP*UR | CGC GGATCC CCCAGGCGGGTATAGAGGT (BamH I) | | This study |  |  |
| *phoP*DF | CGC GGATCC TCAAGCCGATCGATACGGT (BamH I) | | This study |  |  |
| *phoP*DR | CCC AAGCTT GCAGGCTTACCCGCTTGT (Hind III) | | This study |  |  |
| *phoQ*UF | CCG GAATTC CGAGAACATCTCCGACACCAGCAGC (EcoR I) | | This study |  |  |
| *phoQ*UR | CGC GGATCC GCCAGCATGAACAGCACCGCCAGGG (BamH I) | | This study |  |  |
| *phoQ*DF | CGC GGATCC GACTTGGCGGCCGTTACCTACGTCT (BamH I) | | This study |  |  |
| *phoQ*DR | CCC AAGCTT GAGGTCCAGTTCCAGGTCGTCCACA (Hind III) | | This study |  |  |
| *arnB*UF | CCG GAATTC GACGCCGACATCTACGACAC (EcoR I) | | This study |  |  |
| *arnB*UR | CGC GGATCC ACGCAGGACCTGCTCCAC (BamH I) | | This study |  |  |
| *arnB*DF | CGC GGATCC TGCCCAACAGCGAGTGG (BamH I) | | This study |  |  |
| *arnB*DR | CCC AAGCTT AGGTCATGGCGTGGAAGG (Hind III) | | This study |  |  |
|  |  | |  |  |  |
| **Primers used for gene overexpression** | |  |  |  |  |
| PA14_11960F | CGC GGATCC GGACATCAGGACCCGCA (BamH I) | | This study |  |  |
| PA14_11960R | CCC AAGCTT TTGGGTATCGACTGAGCGC (Hind III) | | This study |  |  |
| PA14_11970F | CGC GGATCC TGGTCCCAGCGACAATCC (BamH I) | | This study |  |  |
| PA14_11970R | CCC AAGCTT CAGCCACTCGGGGTTAGC (Hind III) | | This study |  |  |
| PA14_46900F | CGC GGATCC AGCAGAATTCACGCTGCGA (BamH I) | | This study |  |  |
| PA14_46900R | CCC AAGCTT GGGGAACAGACGACAGCCTA (Hind III) | | This study |  |  |
| PA14_50740F | CGC GGATCC TTATCCCTTTCGGAGATGCC (BamH I) | | This study |  |  |
| PA14_50740R | CCC AAGCTT TCGGCGCTACAACGAAAAC (Hind III) | | This study |  |  |
| PA14_52350F | CGC GGATCC TGTCGCGCAAATTCCCAT (BamH I) | | This study |  |  |
| PA14_52350R | CCC AAGCTT CCAGGGCTTCCAGTTTCG (Hind III) | | This study |  |  |
| PA14_52370F | CGC GGATCC GGCCTGACTGGATTGGTGAA (BamH I) | | This study |  |  |
| PA14_52370R | CCC AAGCTT GAAGGAGCAGGGAGCCTGAT (Hind III) | | This study |  |  |
|  |  | |  |  |  |
| **Cloning of genes for LacZ reporter construction** | |  |  |  |  |
| PA14_11970ZF | TCC CCCGGG GCTTTCGATTACCCGCACC (Sma I) | | This study |  |  |
| PA14_11970ZR | CGC GGATCC CAGGGCAAACTAAGGATGGG (BamH I) | | This study |  |  |
| PA14_46900ZF | TCC CCCGGG GCAGACTGGGAGCGATACG (Sma I) | | This study |  |  |
| PA14_46900ZR | CGC GGATCC CCAGCAGGCTCAGGATGAG (BamH I) | | This study |  |  |
| PA14_50740ZF | TCC CCCGGG CGCGAGAAGGGTAGTCGG (Sma I) | | This study |  |  |
| PA14_50740ZR | CGC GGATCC GAAGCAACGATCAAGGCAGA (BamH I) | | This study |  |  |
| PA14_52350ZF | TCC CCCGGG GCTCGCATCATCGCACAT (Sma I) | | This study |  |  |
| PA14_52350ZR | CGC GGATCC TGTTCTGGAACGGGAGCG (BamH I) | | This study |  |  |
|  |  | |  |  |  |
| **Cloning of phoP genes for protein purification** | |  |  |  |  |
| PhoPproF | GATATA CCATGG GCAAACTGCTGGTAGTGGAAGACG (Nco I) | | This study |  |  |
| PhoPproR | GTGGTG CTCGAG CCGGCAGCGCTCGGTGAACAGGTAG (Xho I) | | This study |  |  |
|  |  | |  |  |  |
| **Constructing of *phoP*-Flag gene for ChIP-seq analyzation** | |  |  |  |  |
| P-PhoP-S | TTGTTC AAGCTT TGGCCCAGCCTCTATGCAGGCGACT (Hind III) | | This study |  |  |
| P-PhoP-A | CTGCGC CTGCAG GGTGTTTCTCCGTTCTGCGAGAGTC (Pst I) | | This study |  |  |
| PhoP-flag-S | TGACCG CTGCAG GCCCGCGAGGGCATGCTTCGATGGC (Pst I) | | This study |  |  |
| PhoP-flag-A | CGC GGATCC TTTATCATCATCATCTTTGTAATC TCCGCC CCGGCAGCGCTCGGTGAACAGGTAG (BamH I) | | This study |  |  |
|  |  | |  |  |  |
| **Annealing of DNA probe for EMSA** | |  | | | This study |
| P_PA14_11970_F | TGTCTTTGTAACAGGAAATCCTCGAT**GTTCAG**TCCGAAACCGAGCTGCGCGCCCCAGTTG | | This study |  |  |
| P_PA14_11970_R | CAACTGGGGCGCGCAGCTCGGTTTCGGACTGAACATCGAGGATTTCCTGTTACAAAGACA | | This study |  |  |
| P_PA14_46900_F | GGTTC**GTTCAG**AAATT**GTTCAG**GCTTC**GTTCAG**GCCCG**ATTCAG**GCCGG**GTTCAG**CAGAA | | This study |  |  |
| P_PA14_46900_R | TTCTGCTGAACCCGGCCTGAATCGGGCCTGAACGAAGCCTGAACAATTTCTGAACGAACC | | This study |  |  |
| P_PA14_50740_F | AATCG**ATTCAG**CAATGGTACAGGTTC**GTTCAG**TTTCGACCGAACCCTCCCGCTTTCATCG | | This study |  |  |
| P_PA14_50740_R | CGATGAAAGCGGGAGGGTTCGGTCGAAACTGAACGAACCTGTACCATTGCTGAATCGATT | | This study |  |  |
| P_PA14_52350_F | CATTTGCTGGTGTTC**GTTCAG**CGATG**GTTCAG**CTTGCCGTGGGCATAGTGGCGGCTTGCA | | This study |  |  |
| P_PA14_52350_R | TGCAAGCCGCCACTATGCCCACGGCAAGCTGAACCATCGCTGAACGAACACCAGCAAATG | | This study |  |  |
| IFPF | TACGAATACCGCATCCTCGAATACCTCATGCGGCATCACCAGCAGGTGGTGGCCAAGGAA | | This study |  |  |
| IFPR | TTCCTTGGCCACCACCTGCTGGTGATGCCGCATGAGGTATTCGAGGATGCGGTATTCGTA | | This study |  |  |
|  |  | |  |  |  |
| **Quantitative real-time PCR primers for gene expression level detection** | |  |  |  |  |
| *rpsL*RTF | CAAGCGCATGGTCGACAAGAG | | This study |  |  |
| *rpsL*RTR | ACCTTACGCAGTGCCGAGTTC | | This study |  |  |
| PA14_11970RTF | TACTACCTGGAGGAGAAA | | This study |  |  |
| PA14_11970RTR | GCGTAATACATATAGATGTG | | This study |  |  |
| PA14_11980RTF | GGGCTGGTGAACTATTTC | | This study |  |  |
| PA14_11980RTR | GAAGCGTTTAAGTTCGGTC | | This study |  |  |
| PA14_21860RTF | ATGAACTCTGAATGGATCTGG | | This study |  |  |
| PA14_21860RTR | CAGCAGGTGACCGTAGAA | | This study |  |  |
| PA14_21870RTF | ATCCAGCAATGCCTCGAA | | This study |  |  |
| PA14_21870RTR | TAGCCGTAGAGATCGTTGA | | This study |  |  |
| PA14_46900RTF | GACGGCGACTTCTGGTAC | | This study |  |  |
| PA14_46900RTR | CTGGTGGTTGTTGTGTTC | | This study |  |  |
| PA14_46910RTF | ACGCTGAAGAAGATCAAG | | This study |  |  |
| PA14_46910RTR | GAGGTAGGAGAAGGGAAT | | This study |  |  |
| PA14_50740RTF | TGATCGTTGCTTCCTTCA | | This study |  |  |
| PA14_50740RTR | TGTAGGTATCGCCGGTGA | | This study |  |  |
| PA14_50750RTF | ATGGACTATTTCATCATCGTCGT | | This study |  |  |
| PA14_50750RTR | GCGGATGCGCCAGTAGAT | | This study |  |  |
| PA14_52340RTF | CTGTTCTGCGGTTCCCTG | | This study |  |  |
| PA14_52340RTR | CAGGCTCCAGGACTCTTTC | | This study |  |  |
| PA14_52350RTF | TACGCTACCGACTGTATT | | This study |  |  |
| PA14_52350RTR | GGAAAGGCTGGCAAATAG | | This study |  |  |
| *pilY1*RTF | TATACCTACAATGCTCTTCCTAC | | This study |  |  |
| *pilY1*RTR | GATGCGGTTGCGATAGTA | | This study |  |  |

^a^ Primer designation as follows: F, forward; R, reverse; U, upstream of indicated gene; D, downstream of indicated gene; Z, *lacZ*Ω; IFP, **i**nternal **f**ragment of ***p****hoP* open reading frame; RT, real-time PCR. ^b^ The underlined sequences represent restriction enzymes cutting sites. The sequence of Flag tag is indicated by a dotted line. The colored letters represent the PhoP binding motifs.
