# Supplementary material for: Identification of Novel phoP-phoQ Regulated Genes that Contribute to Polymyxin B Tolerance in Pseudomonas aeruginosa
Source: Microorganisms. 2021 Feb 9;9(2):344. doi: 10.3390/microorganisms9020344 (PMC7916210; doi:10.3390/microorganisms9020344)
Supplement: Supplementary file 1 [file microorganisms-09-00344-s001.zip › Supplementary materials/Table S4 - Bacterial susceptibilities to polymyxin B in CA-MHB.docx]

**Table S4. Bacterial susceptibilities to polymyxin B in CA-MHB.**

| **Strain** | **MIC (μg/mL)** |
| --- | --- |
| PA14 | 0.3125 |
| Δ*phoP* | 0.1563 |
| Δ*papP*::Tn | 0.3125 |
| Δ*mpl*::Tn | 0.3125 |
| ΔPA14_11980::Tn | 0.3125 |
| Δ*pagP*::Tn | 0.3125 |
| Δ*slyB*::Tn | 0.3125 |
| ΔPA14_52340::Tn | 0.3125 |
| Δ*ppgS*::Tn | 0.3125 |
| Δ*ppgH*::Tn | 0.3125 |
| Δ*papP* | 0.3125 |
| Δ*mpl* | 0.3125 |
| Δ*pagP* | 0.3125 |
| Δ*slyB* | 0.3125 |
| Δ*ppgS* | 0.3125 |
| Δ*ppgH* | 0.3125 |
| Δ5 ^a^ | 0.3125 |
| Δ6 ^b^ | 0.1563 |

^a^ Δ5, Δ*papP*Δ*mpl*Δ*slyB*Δ*ppgS*Δ*ppgH*; ^b^ Δ6, deletion of *pagP* in Δ5.
